# Supplementary material for: Implications for human odor sensing revealed from the statistics of odorant-receptor interactions
Source: PLoS Comput Biol. 2018 May 21;14(5):e1006175. doi: 10.1371/journal.pcbi.1006175 (PMC5983876; doi:10.1371/journal.pcbi.1006175)
Supplement: S1 Text — We provide details for two observations that were mentioned in the main text: (i) deviation from the Michaelis-Menten kinetics, (ii) binarized cellular response to odor concentration. (PDF) [file pcbi.1006175.s001.pdf]

Supporting Information for:

Implications for human odor sensing revealed from the statistics of odorant-receptor interactions

Ji Hyun Bak[1], Seogjoo J. Jang[2,3], Changbong Hyeon[1,*]

1 School of Computational Sciences, Korea Institute for Advanced Study, Seoul 02455, Korea
2 Department of Chemistry and Biochemistry, Queens College, City University of New York, 65-30 Kissena Boulevard, Queens, New York 11367, United States
3 PhD programs in Chemistry and Physics, and Initiative for Theoretical Sciences, Graduate Center, City University of New York, 365 Fifth Avenue, New York, New York 10016, United States

* hyeoncb@kias.re.kr

S1 Text

Deviation from the Michaelis-Menten kinetics

The deviation of the odor response from the Michaelis-Menten (MM) form of Eq 2, namely the fitting of Hill curve with $H \neq 1$, could originate from multiple sources. Here we describe three possible scenarios where we can observe such deviation, and offer relevant quantitative analyses.

A cooperative activation of oligomerized receptors

Let us consider the situation where the receptors form a dimer, resulting in two binding sites to which a specific type of odorant can bind. Equilibrium constants for the two binding sites, given as $K_1 = [\mathrm{OR}][\mathrm{O}]/[\mathrm{OR}\cdot\mathrm{O}_1]$ and $K_2 = [\mathrm{OR}\cdot\mathrm{O}_1][\mathrm{O}]/[\mathrm{OR}\cdot\mathrm{O}_2]$, yield the following fractional occupancy of the dimeric complex, which is translated to an odorant concentration ($C_O = [O]$)-dependent OR activity:

$$f = \frac{[\mathrm{OR}\cdot\mathrm{O}_1] + 2[\mathrm{OR}\cdot\mathrm{O}_2]}{2\mathrm{OR}_o} = \frac{\frac{C_O}{K_1} + 2\frac{C_O^2}{K_1K_2}}{2\left(1 + \frac{C_O}{K_1} + \frac{C_O^2}{K_1K_2}\right)}. \tag{S1}$$

where $\mathrm{OR}_o = [\mathrm{OR}] + [\mathrm{OR}\cdot\mathrm{O}_1] + [\mathrm{OR}\cdot\mathrm{O}_2]$. In this case, the Hill coefficient is obtained using Eq 9 as:

$$n_H = \frac{4}{2 + \sqrt{K_2/K_1}}. \tag{S2}$$

If the two binding sites have positive cooperativity, we have $K_2 \leq K_1$, which gives rise to $1 \leq n_H \leq 2$.

The amplification of sensitivity through the signal cascades via GTP hydrolysis

Even in the absence of allosteric cooperativity, a highly sigmoidal, switch-like response can arise from a reversible covalent modification along the signaling pathway. The reversible covalent modification is exemplified by the signaling processes such as phosphorylation/dephosphorylation and GDP/GTP exchange accompanied with GTP hydrolysis, whose effect on the sensitivity of signaling is a well studied issue [1–3] since the seminal work by Goldbeter and Koshland [4].

In the context of our study, the amount of GDP-bound G-protein (G_D) in response to the stimuli (odorant) defines the olfactory activity. Although our minimal kinetic model for odorant-OR kinetics did not explicitly take into account the effect of GDP/GTP exchange in G-protein and recycling of GDP from GTP hydrolysis, such mechanistic details can modulate the sensitivity of olfactory signaling and consequently make the Hill coefficient deviate from unity. Here we provide an overview of amplified sensitivity through covalent modification by explicitly using the terminologies for the OR signaling.

When OR is in the active form ($\mathrm{OR_G}$ in Scheme 1), it catalyzes the exchange of G_D into G_T; on the other hand, the GTPase activating protein (GAP) hydrolyzes the GTP in G_T to produce G_D back.

$$\mathrm{OR} \underset{k_{-1}}{\overset{k_1}{\rightleftharpoons}} \mathrm{OR}\cdot\mathrm{G}_D \underset{k_{-2}[\mathrm{D}]}{\overset{k_2[\mathrm{T}]}{\rightleftharpoons}} \mathrm{OR} + G_T$$
$$\mathrm{GAP} \underset{k_{-3}}{\overset{k_3}{\rightleftharpoons}} \mathrm{GAP}\cdot G_T \underset{k_{-4}[P_i]}{\overset{k_4}{\rightleftharpoons}} \mathrm{GAP} + G_D \tag{S3}$$

The amount of G_D in the pool of G-proteins ($G_o = [G_D] + [G_T]$) is the key quantity that determines the G-protein signaling. The variation of G_D is given by the difference of the incoming and outgoing currents, J_+ and J_-:

$$\begin{aligned}\partial_t[G_D] &= -J_- + J_+ \\ &= -\frac{\frac{V_1[G_D]}{K_1} - \frac{V_1^*[G_T]}{K_1^*}}{1 + \frac{[G_D]}{K_1} + \frac{[G_T]}{K_1^*}} + \frac{\frac{V_2^*[G_T]}{K_2^*} - \frac{V_2[G_D]}{K_2}}{1 + \frac{[G_T]}{K_2^*} + \frac{[G_D]}{K_2}}\end{aligned} \tag{S4}$$

where new notations were introduced for the maximum rates (V's) and Michaelis constants (K's), associated with GDP→GTP exchange in G-protein by OR ($V_1 = k_2(\mathrm{OR})_o[T]$, $K_1 = \frac{k_2[T]+k_{-1}}{k_1}$); reverse exchange of GTP→ GDP in G-protein ($V_1^* = k_{-1}(\mathrm{OR})_o$, $K_1^* = \frac{k_2[T]+k_{-1}}{k_{-2}[D]}$); GTP hydrolysis by GAP ($V_2^* = k_4(\mathrm{GAP})_o$, $K_2 = \frac{k_4+k_{-3}}{k_{-4}[P_i]}$); and GTP synthesis ($V_2 = k_{-3}(\mathrm{GAP})_o$, $K_2^* = \frac{k_4+k_{-3}}{k_3}$). It is useful to define three dimensionless parameters:

$$\theta = \frac{V_1/K_1}{V_2^*/K_2^*}, \qquad \mu = \frac{V_2/K_2}{V_2^*/K_2^*}, \qquad \gamma = \frac{k_1k_2k_3k_4[T]}{k_{-1}k_{-2}k_{-3}k_{-4}[D][P_i]} \tag{S5}$$

where γ is associated with the net non-equilibrium chemical driving force of G-protein signaling pathway in the form of $\Delta\psi = -k_BT\log\gamma$. Note that $\Delta\psi = 0$ (or $\gamma = 1$) at equilibrium.

From the steady state solution $\partial_t[G_D] = 0$ (Eq S4), we can calculate the activity $f = \frac{[G_D]^{ss}}{[G_D]^{ss}+[G_T]^{ss}} = \frac{[G_D]^{ss}}{G_o}$ as

$$\theta = \frac{\mu\gamma[\mu - (\mu+1)f]\left[f\left(1 - \frac{K_1}{K_1^*}\right) - \left(1 + \frac{K_1}{G_o}\right)\right]K_2^*}{[\mu\gamma - (\mu\gamma+1)f]\left[f\left(1 - \frac{K_2^*}{K_2}\right) + \frac{K_2^*}{K_2}\left(1 + \frac{K_2}{G_o}\right)\right]K_1}. \tag{S6}$$

Recall that the neuronal response is directly determined by the amount of G_D ($\sim f$), which is in turn dependent on the amount of active OR ($\sim \theta$). Therefore the sharpness of response $f(\theta)$ is characterized by the effective Hill coefficient, which is once again obtained using Eq 9:

$$n_H = \left[\frac{\mu(\gamma-1)}{(\mu\gamma-1)(1-\mu)} - \frac{1 - \frac{K_1}{K_1^*}\frac{K_2^*}{K_2} + \left\{ \frac{K_1}{G_o}\left(1-\frac{K_2^*}{K_2}\right) + \frac{K_2^*}{G_o}\left(1-\frac{K_1}{K_1^*}\right)\right\}}{\left(1+\frac{K_1}{K_1^*}+2\frac{K_1}{G_o}\right)\left(1+\frac{K_2^*}{K_2}+2\frac{K_2^*}{G_o}\right)} \right]^{-1}. \quad \text{(S7)}$$

In summary, when GDP/GTP exchange and GTP hydrolysis are explicitly taken into account, the expression of $f(\theta)$ differs from the conventional type of MM expression, and the Hill coefficient evaluated using Eq 9 depends on the parameters μ, γ, $K_{1,2}$, $K^*_{1,2}$, and G_o. Some limiting conditions greatly simplify Eqs S6 and S7:

(i) If the affinities of G_D to OR and G_T to GAP are sufficiently high ($K_1/K_1^* \ll 1$, $K_2^*/K_2 \ll 1$) that the overall reaction current associated with the production of G_D is positive ($J_+ - J_- \gg 0$), and if the G-protein level is below K_1 and K_2^* ($K_1/G_o \gg 1$, $K_2^*/G_o \gg 1$), the expressions for f and n_H are simplified as:

$$f = \frac{\theta+\mu}{\theta+\mu+\theta/\mu\gamma+1}; \qquad n_H = \frac{(\mu\gamma-1)(1-\mu)}{\mu(\gamma-1)}. \quad \text{(S8)}$$

(ii) In addition to the aforementioned condition of high affinities of G_D to OR and G_T to GAP, if the reversibility of catalytic step is abandoned ($\mu = 0$) together with high chemical driving force imposed by a far-from-equilibrium balance of GTP versus GDP ($\gamma \gg 1$), the activity f is given as

$$\frac{V_1}{V_2^*} = \frac{f(1-f+K_1/G_o)}{(1-f)(f+K_2^*/G_o)}. \quad \text{(S9)}$$

In this case, the Hill coefficient is obtained from $n_H = 4\left(\frac{\partial f}{\partial \log V_1}\right)_{f=1/2}$

$$n_H = \frac{(1+\frac{2K_1}{G_o})(1+\frac{2K_2^*}{G_o})}{\frac{K_1}{G_o}+\frac{K_2^*}{G_o}+\frac{4K_1K_2^*}{G_o^2}}. \quad \text{(S10)}$$

Note that the response is highly sensitized ($n_H \gg 1$) for K_1/G_o, $K_2^*/G_o \ll 1$. This corresponds to the Goldbeter-Koshland formula for zeroth-order ultrasensitivity [3, 4].

Inhomogeneity of the odorant-OR kinetics

Finally, we explore the case when there is inhomogeneity in the parameters for the odorant-OR kinetics. For example, consider the following MM-type hyperbolic activity function:

$$f(\theta;\alpha) = \frac{\alpha\theta}{1+\alpha\theta}. \quad \text{(S11)}$$

Suppose that the parameter α has disorder around its mean value α_0 such that $\alpha = \alpha_0 + \delta\alpha$, $|\delta\alpha| \ll |\alpha|$ where $\delta\alpha$ is a Gaussian random variable satisfying $\delta\alpha \in \mathcal{N}(0, \sigma_\alpha^2)$, then the above function is approximated up to the second order of $\delta\alpha$ as follows:

$$f(\theta;\alpha) \approx f(\theta;\alpha_0) + f'(\theta;\alpha_0)\delta\alpha + \frac{1}{2}f''(\theta,\alpha_0)(\delta\alpha)^2 \quad \text{(S12)}$$

Averaging over the inhomogeneity in α with $\langle\delta\alpha\rangle = 0$ and $\langle(\delta\alpha)^2\rangle = \sigma_\alpha^2$ leads to

$$f \equiv \langle f(\theta,\alpha)\rangle \approx \frac{\alpha_0\theta}{1+\alpha_0\theta} - \frac{(\alpha_0\theta)^2}{(1+\alpha_0\theta)^3}\,\epsilon_\alpha^2 \tag{S13}$$

where $\epsilon_\alpha \equiv \sigma_\alpha/\alpha_0 \ll 1$. From Eq 9, $n_H = 1$ for $\epsilon_\alpha = 0$ as expected, and $n_H \approx 0.94 < 1$ for $\epsilon_\alpha = 0.5$. The effect is relatively minor compared to the previous two cases, as long as we are in the small fluctuation regime $\epsilon_\alpha \ll 1$. Nonetheless, the inhomogeneity in parameter is still a possible source of the deviation from MM kinetics. It is interesting to note that the inhomogeneity in kinetic parameter always de-sensitizes the response ($n_H \lesssim 1$).

Binarized cellular response to odor concentration

Here we outline a simple argument for the effective binarization of cellular responses to odor concentration. Since $p(C_O) \equiv (S(C_O) - B)/\delta S_{\max}$ is the activation probability for a single OR at an odorant concentration C_O, the probability of having ℓ out of L ORs activated is described by a C_O-dependent binomial distribution: $P(\ell; C_O, L) = \binom{L}{\ell}\, p(C_O)^\ell\,(1-p(C_O))^{L-\ell}$. For large number of receptors, satisfying L, $Lp(C_O)$, $L(1-p(C_O)) \gg 1$, the binomial distribution is approximated to a normal distribution with a mean $\bar{\ell}(C_O) = Lp(C_O)$ and a variance $\sigma_\ell^2(C_O) = Lp(C_O)(1-p(C_O))$. Then, the probability distribution of having ℓ out L receptors activated is

$$P(\ell/L; C_O) \simeq \frac{1}{\sqrt{2\pi\epsilon_\ell^2}}\, e^{-(\ell/L-\bar{\ell}/L)^2/2\epsilon_\ell^2} \tag{S14}$$

where $\epsilon_\ell^2 = \sigma_\ell^2/L^2 = p(1-p)/L \le (4L)^{-1}$. Thus, the fluctuation of the fraction of activated ORs, $\langle(\delta\ell/L)^2\rangle$, is suppressed if the population size (L) is large. For $L \sim 2.5 \times 10^4$ [5], the size of this fluctuation is limited to less than 0.6 %: $\epsilon_\ell \lesssim 1/\sqrt{L} < 6 \times 10^{-3}$.

Meanwhile, the membrane potential V_m of a neuron is modulated by changes in the ratio of ion concentrations inside and outside the membranes, which is related to the fraction of open and closed ion channels (or to the fraction of activated and inactivated ORs) (Fig 1a), such that $V_m \sim \log\left(\frac{C_{\text{ion}}^{(\text{out})}}{C_{\text{ion}}^{(\text{in})}}\right) \sim \log\left(\frac{1-\ell/L}{\ell/L}\right) \equiv g(\ell/L)$ [6,7]. The first relation is none other than the Nernst equation. Therefore, in the small noise limit one can map C_O to $\bar{\ell}$ and $\bar{\ell}$ to $\bar{V}_m$, or vice versa, using the monotonic relationships $\bar{\ell}/L = p(C_O)$ and $\bar{V}_m = g(\bar{\ell}/L)$, respectively. Note that the generation of a neural spike (action potential) is in general evoked when the membrane potential exceeds a threshold V_m^θ [6,7]; thus V_m^θ can be effectively related to a threshold in ℓ/L, or to C_O, such that $V_m^\theta = g(\ell^\theta/L)$ and $\ell^\theta/L = p(C_O^\theta)$ (See Fig A).

For a given threshold potential, the firing probability of a neuron corresponds to the probability that more than ℓ^θ receptors are activated, and it can be written as:

$$\begin{aligned} F(C_O; \ell^\theta, L) &= \int_{\ell^\theta/L}^{1} P(\ell/L; C_O)\, d(\ell/L) \\ &\simeq \frac{1}{2}\,\text{erfc}\left[\frac{\ell^\theta/L - \bar{\ell}(C_O)/L}{\sqrt{2}\epsilon_\ell(C_O)}\right], \end{aligned} \tag{S15}$$

where erfc is the complimentary error function, which is approximated to $\text{erfc}(z) \simeq 1 + \text{sign}(z)$ for $|z| \gtrsim 2$. For large L, $\epsilon_\ell \sim 1/\sqrt{L}$, which increases the precision of ℓ/L for a given C_O. The size of the argument of the erfc in Eq S15 is greater than $\sqrt{2L}\,|\ell^\theta/L - \bar{\ell}/L|$; for large L, it is clearly in the $|z| \gg 2$ regime. Thus, the firing probability can be approximated to the step function as in Eq 17.

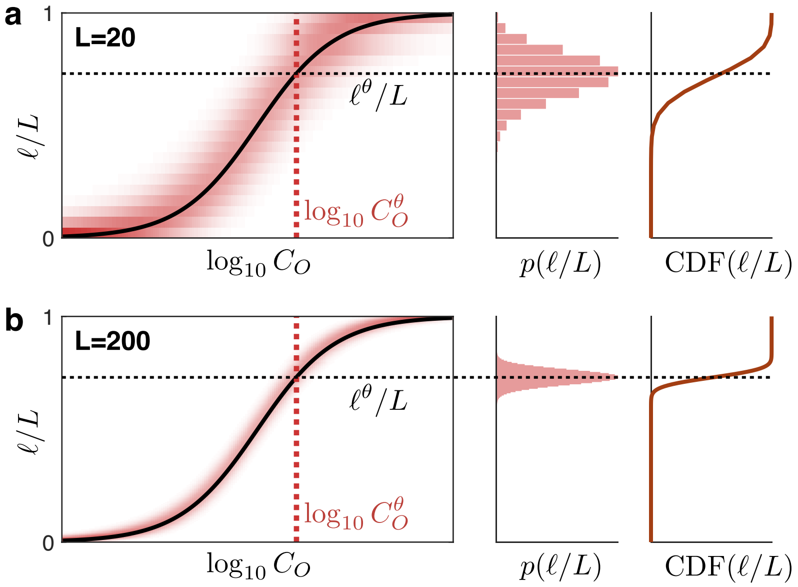

Fig A. Illustration for the binarized cellular response argument. The sharpness of the response curve depends on the receptor copy number L (**a.** $L = 20$, **b.** $L = 200$).

References

1. Qian H. Thermodynamic and kinetic analysis of sensitivity amplification in biological signal transduction. Biophys Chem. 2003;105:585–593.
2. Beard DA, Qian H. Chemical Biophysics: Quantitative Analysis of Cellular Systems. Cambridge University Press; 2008.
3. Qian H. Phosphorylation Energy Hypothesis: Open Chemical Systems and Their Biological Functions. Annu Rev Phys Chem. 2007;58:113–142.
4. Goldbeter A, Koshland, Jr DE. An amplified sensitivity arising from covalent modification in biological systems. Proc Natl Acad Sci U S A. 1981;78:6840–6844.
5. Bhandawat V, Reisert J, Yau KW. Signaling by olfactory receptor neurons near threshold. Proc Natl Acad Sci U S A. 2010;107(43):18682–18687.
6. Kandel ER, Schwartz JH, Jessell TM, Siegelbaum SA, Hudspeth AJ, editors. Principles of Neural Science. 5th ed. McGraw Hill; 2013.
7. Luo L. Principles of neurobiology. Garland Science; 2016.
